# Der p 1-based immunotoxin as potential tool for the treatment of dust mite respiratory allergy

**DOI:** 10.1038/s41598-020-69166-w

**Published:** 2020-07-23

**Authors:** Rodrigo Lázaro-Gorines, Juan Carlos López-Rodríguez, Sara Benedé, Miguel González, Cristobalina Mayorga, Lothar Vogel, Álvaro Martínez-del-Pozo, Javier Lacadena, Mayte Villalba

**Affiliations:** 1 Biochemistry and Molecular Biology Department, Chemical Sciences Faculty, Complutense University of Madrid, Av. Complutense w/n, 28040 Madrid, Spain; 2 Allergy Research Laboratory, IBIMA, Hospital Regional Universitario de Málaga, UMA, Málaga, Spain; 3 U.G.C. Allergy, IBIMA, Hospital Regional Universitario de Málaga, UMA, Málaga, Spain; 4 Division of Allergology, Paul-Ehrlich-Institut, Langen, Germany

**Keywords:** Antigen presentation, Allergy, Asthma, Recombinant protein therapy

## Abstract

Immunotoxins appear as promising therapeutic molecules, alternative to allergen-specific-immunotherapy. In this work, we achieved the development of a protein chimera able to promote specific cell death on effector cells involved in the allergic reaction. Der p 1 allergen was chosen as cell-targeting domain and the powerful ribotoxin α-sarcin as the toxic moiety. The resultant construction, named proDerp1αS, was produced and purified from the yeast *Pichia pastoris*. Der p 1-protease activity and α-sarcin ribonucleolytic action were effectively conserved in proDerp1αS. Immunotoxin impact was assayed by using effector cells sensitized with house dust mite-allergic sera. Cell degranulation and death, triggered by proDerp1αS, was exclusively observed on Der p 1 sera sensitized-humRBL-2H3 cells, but not when treated with non-allergic sera. Most notably, equivalent IgE-binding and degranulation were observed with both proDerp1αS construct and native Der p 1 when using purified basophils from sensitized patients. However, proDerp1αS did not cause any cytotoxic effect on these cells, apparently due to its lack of internalization after their surface IgE-binding, showing the complex in vivo panorama governing allergic reactions. In conclusion, herein we present proDerp1αS as a proof of concept for a potential and alternative new designs of therapeutic tools for allergies. Development of new, and more specific, second-generation of immunotoxins following proDerp1αS, is further discussed.

## Introduction

Allergy or type I hypersensitivity reactions are widely described as a loss of tolerance to certain harmless exogenous antigens, such as airborne allergens, foods or insect venoms. Allergies prevalence currently reaches the 30% among the population in the industrialized countries, experiencing an alarming increment over the last few decades^1^. Allergies cause a substantial social and economic impact, being the development of new treatments a crucial goal for biomedical research. Common symptoms of allergies, such as asthma and rhinitis, share a strong T-helper type 2 (Th2) immune polarisation, in combination with a significant decreased activity of the IFNγ-secreting Th1 cells. Because of this shift, several active mediators are released (e.g. histamine, leukotrienes and prostaglandins, among others) by basophils and mast cells, ultimately resulting in the main immune-pathological features of the allergic response^2,3^.

Despite the well accepted fact that our understanding of the mechanisms underlying allergies has dramatically improved lately, we are still far from developing an effective therapy because of the multifactorial origin of this pathology. Accordingly, oral tolerance induction has shown to be highly effective in patients at early ages, but it dramatically fails once most immune responses have already been established. Within this same idea, allergen-specific-immunotherapy (AIT) arises as a unique alternative^4,5^, but carries powerful limitations that completely offset its clinical benefits. These limitations include: the variable quality of the vaccine components, its unequally effectiveness within patients, the onset of certain adverse side-effects, and its limited action at the basis of the Th2-driven effector phase^6,7^. Finally, the exact mechanisms underlying the success, or failure, of AIT are still under intense debate.

In this context, immunotoxins arise as a promising approach beyond cancer treatment, revealing themselves as promising candidates against inflammatory diseases such as allergies, mainly because of their specific ability to promote cell death. Immunotoxins are chimeric molecules that combine a targeting domain-usually antibody based-fused to a toxin domain providing the molecule with its cytotoxic activity^8,9^. Their mode of action comprises three main steps: (1) recognition of the antigen by the targeting domain; (2) cell internalization of the complex; and (3) subsequent intracellular release of the active toxic domain which catalytically causes target cell death^10,11^.

Regarding type I hypersensitivity reactions, the obvious suitable targeting domain for immunotoxin development would be the inclusion of an allergen, specifically directed to IgE presented on the cell surface of effector (basophils, mast cells) and B cells^12–14^. In this sense, an early example of this strategy has been used in animal models. Ovalbumin (OVA)-sensitized BALB/c mice were treated with an OVA-diphtheria toxin chimera, which resulted in protection from anaphylactic shock after OVA re-challenge^15^ and simultaneous depletion of OVA-specific IgE and IgG1 levels but increased IgG2a^16^.

In this work we report the development of a different design using the prevalent house dust mite (HDM) allergen Der p 1, as targeting domain. Der p 1-sensitization accounts for more than 80% in mite-allergic subjects, affecting to more than 20% of the global population and up to 85% of asthmatics allergic to HDM^17^. Der p 1 is a papain-like cysteine protease which is synthetized as a 34 kDa proenzyme (proDer p 1), consisting of a cysteine protease domain (222 residues) and an N-terminal pro-peptide (80 amino acids) that blocks its proteolytic activity^18,19^. Der p 1 pro-peptide also functions as an intramolecular chaperone ensuring its correct folding and extracellular secretion^20^. Importantly, this pro-sequence has been used for recombinant Der p 1 production both in tobacco plants and in the yeast *P. pastoris*^21–23^. Regarding the toxic domain, we have chosen the highly specific ribotoxin α-sarcin, an extracellular fungal RNase secreted by *Aspergillus giganteus*, which specifically cleaves a unique single bond of the larger rRNA located at the so-known Sarcin Ricin Loop (SRL)^24^. This cleavage impairs ribosome function and, thereby, causes protein biosynthesis inhibition, leading to programmed cell death by apoptosis^25,26^. α-Sarcin is the most powerful ribotoxin candidate known so far according to its functional properties and previously described antitumoral therapeutic applications^27–31^.

Herein we present the design and characterization of an innovative allergen-based immunotoxin, consisting in the recombinant fusion of the allergen proDer p 1 and α-sarcin, representing a proof of concept for studying the potential of these molecules as allergy treatment. The chimeric construct, named proDerp1αS, was produced in the yeast *P. pastoris* and evaluated on humanized basophil-like cells (humRBL-2H3) sensitized with Der p 1 allergic patients’ sera. Potential non-specific cytotoxicity of the construct was also evaluated in other cell lines such as HeLa, Calu-3, LAD2 and Raw 264.7. Finally, the biological relevance of proDerp1αS was reported in terms of activation and cytotoxicity against ex vivo basophils from Der p 1 allergic patients and non-allergic individuals.

## Results

### proDerp1αS generation, production and purification

**Figure 1:**
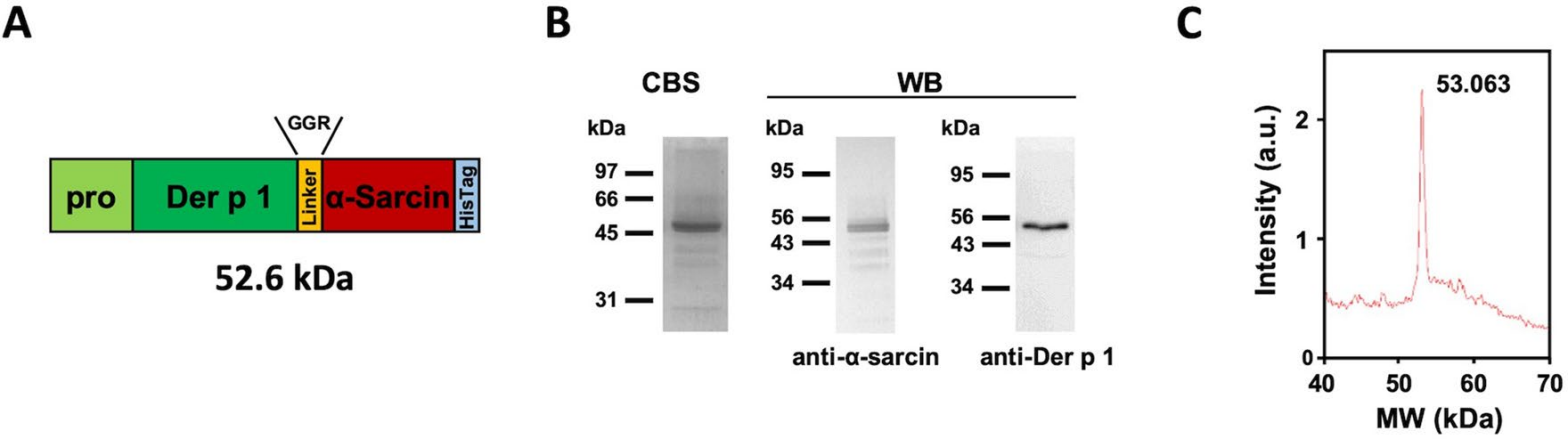
Domain arrangement of proDerp1αS construct, along with SDS-PAGE, Western blot and mass spectrometry analysis of the purified immunotoxin. (**A**) Schematic representation of proDerp1αS cDNA, highlighting its structural and functional motifs, alongside its theoretical molecular mass. 3D-model structure of proDerp1αS is included as Figure S1. (**B**) Coomassie blue stained SDS-PAGE (CBS) and Western blot analysis using rabbit anti-α-sarcin (left) and anti-α-Der p 1 antisera (right). CBS protein molecular weight standards correspond to Bio-Rad Unstained SDS-PAGE low range Standards; while the prestained Bio-Rad Precision. Plus Dual Color Standards (Bio-Rad, Hercules, CA, USA) were used for Western blot. Images correspond to full-length gels and blots acquired and analyzed using the Gel Doc XR Imaging System and Quantity One 1-D analysis sofware (BioRad) or ChemiDoc-It (UVP) and VisionWorks LS, respectively.Full-length blots/gels are presented in Supplementary Figure S2. (**C**) Mass spectrometry analysis spectrum showing the corresponding peak to proDerp1αS construct. The difference between theoretical and empirical masses agrees with the four N-terminal extra amino acids of α-factor secretion signal that could remain after its processing by kex2 protease.

The corresponding proDer p 1 and α-sarcin cDNAs were fused by means of a glycine-glycine-arginine (GGR) linker to produce the proDerp1αS construction (Fig. 1A and S1). This construction was then cloned in pPICZαA, downstream of the α-factor secretion signal sequence within the plasmid, and successfully produced in *P. pastoris* yeast extracellular culture medium (Fig. 1B, C). The protein was purified, after extensive dialysis against 50 mM sodium phosphate buffer, 0.1 M NaCl, pH 7.5, by means of a Ni^2+^-NTA affinity chromatography, taking advantage of its C-terminal six histidine extension (Fig. 1A, in light blue). The purified protein, analyzed by SDS-PAGE, showed a molecular mass in agreement with the theoretical 52.6 kDa expected for the chimeric construct (Fig. 1B). The purification yield of the protein was 1 mg/L of induction medium.

### Structural characterization of proDerp1αS

The purified protein was specifically recognized by both anti-α-sarcin and anti-Derp1 rabbit antisera by Western blot immunodetection, proving the chimeric composition of the construct (Fig. 1B). Furthermore, MALDI-TOF mass spectrometry for proDerp1αS reported a single peak which corresponds to the atomic mass expected for the non-processed whole protein plus the four N-terminal amino acids left after kex2 protease processing of α-factor secretion signal (Fig. 1C), indicating thereby that Der p 1 pro-peptide was not removed.

### Enzymatic characterization

**Figure 2:**
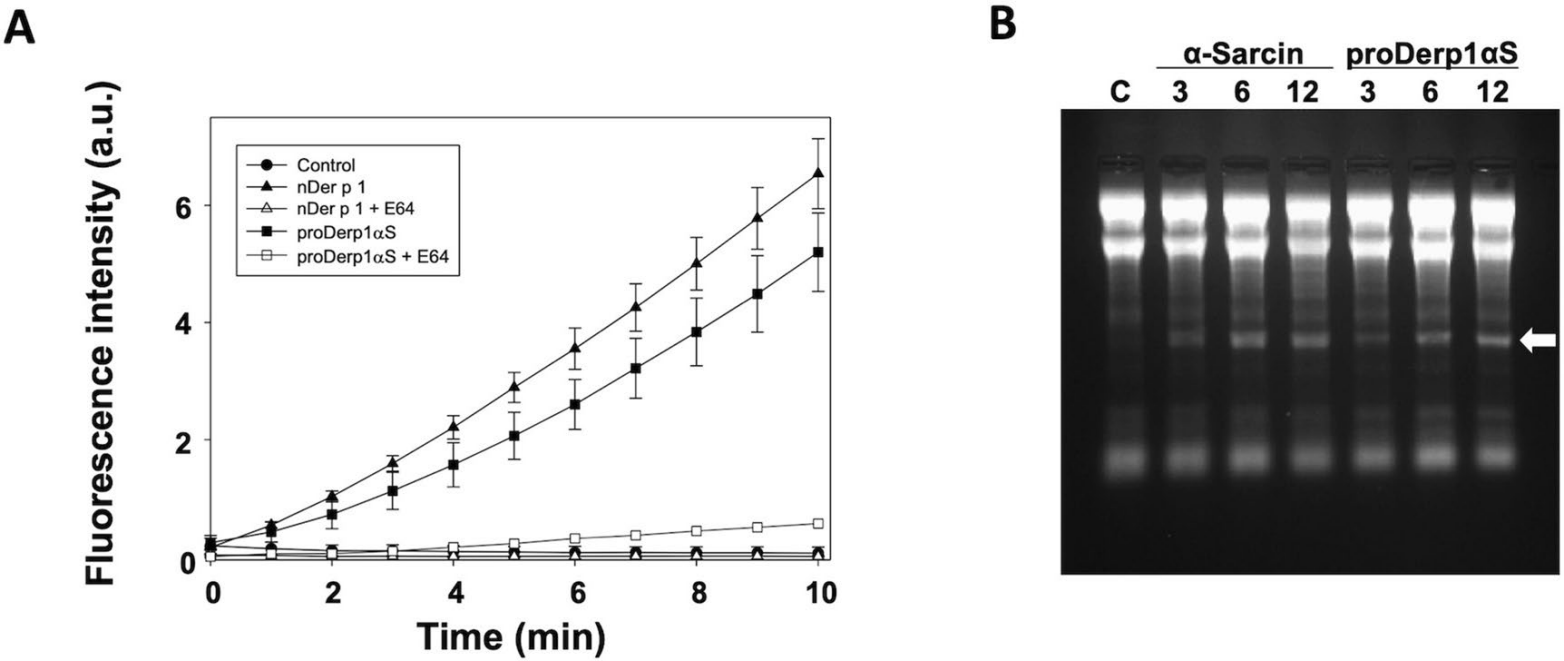
In vitro functional characterization of the allergen and toxin domains. (**A**) Comparison between nDer p 1 and proDerp1αS cysteine protease activities by mean of Boc-QAR-AMC fluorogenic peptide assay, in the absence or presence of E64 cysteine protease inhibitor. (**B**) Rabbit reticulocytes assays was used for testing the ribonucleolytic activity of α-sarcin within proDerp1αS. α-Fragment release, highlighted by a white arrow, indicates the specific SRL cleavage. Equimolar amounts (3, 6, 12 pmol) of α-sarcin and immunotoxin were tested and compared to ribotoxin-free control, C.

As part of the functional characterization, Der p 1-cysteine protease activity of the proDer p 1-based immunotoxin was assayed. The Boc-QAR-AMC fluorogenic peptide assay showed that proDerp1αS was an active protease although displaying a slightly lower specific activity than nDer p 1 (Fig. 2A). The activity of both proteins (native and chimeric) was indeed specific, being completely abolished by the presence of the well-known E64 cysteine-protease inhibitor. Regarding the cytotoxic domain, proDerp1αS retained the highly specific ribonucleolytic activity of α-sarcin against ribosomes, causing the release of the characteristic rRNA α-fragment in a very similar extent to the free fungal wild-type α-sarcin (Fig. 2B).

### IgE recognition by HDM allergic human sera

**Figure 3:**
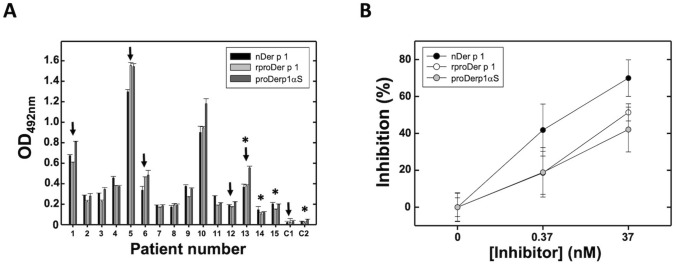
Human serum IgE recognition assays. (**A**) nDer p 1, rproDer p 1 and proDerp1αS comparative detection by serum IgE of Der p 1-positive allergic patients (#1–15) and non-allergic (C1, C2) individuals by mean of indirect ELISA. Black arrows indicate selected sera for in vitro humRBL-2H3 degranulation and viability assays. Asterisks indicate selected patients for BAT and basophils cytotoxicity assays. (**B**) Inhibition of serum binding to nDer p 1 by rproDer p 1 (open circles), the derived immunotoxin chimera (grey circles) and using the allergen nDer p 1(black circles) as a control of maximum inhibition. Graph representation shows the resulting media ± standard deviation for three sera (#1, 3, 10). All experiments were done by duplicate.

Considering the strategy designed and the mechanism of action expected for proDerp1αS on effector cells, the IgE-epitopes binding ability of the allergen within the chimera was analyzed. With this purpose, HDM-allergic sera from human patients were assayed for the comparatively recognition of proDerp1αS, natural Der p 1 (nDer p 1) and recombinant form of proDer p 1 (rproDerp1) in identical conditions (Fig. 3). Allergen-coated indirect enzyme-linked immunosorbent assays (ELISA) using individual Der p 1 allergic and non-allergic patients’ sera showed practically identical IgE recognition behavior against the three proteins in all cases tested (n = 15), controls included (C1 and C2) (Fig. 3A). Therefore, these results showed how the presence of α-sarcin did not impair the ability of specific IgE to recognize the chimeric proDerp1αS construct. Regarding inhibition ELISA assays, rproDer p 1 and proDerp1αS showed behaviors that, although corresponding to lower inhibition values than nDer p 1, were within the experimental error of these assays (Fig. 3B).

### Cell toxicity

**Figure 4:**
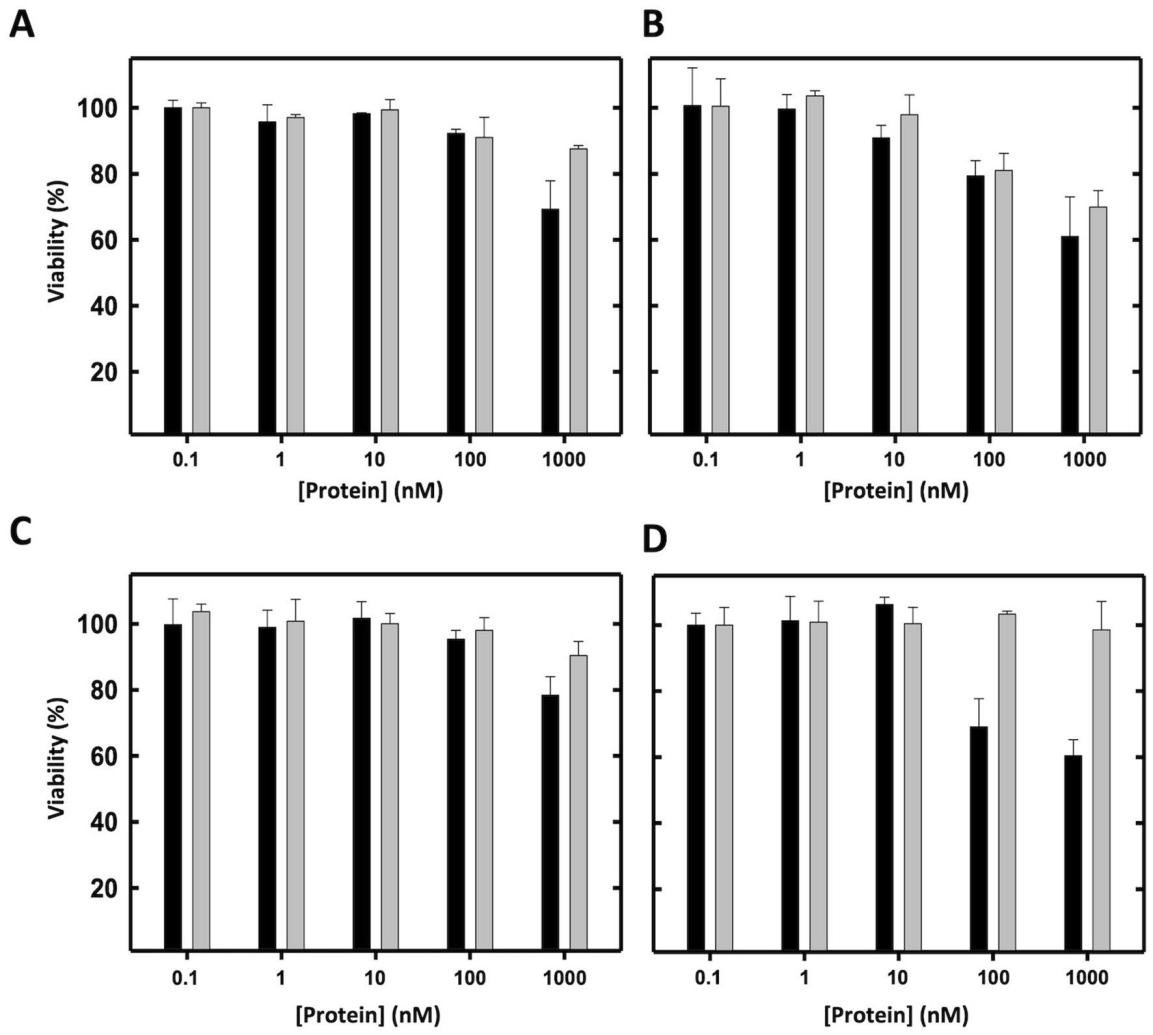
Viability inhibition assays comparing α-sarcin and proDerp1αS cytotoxic activities. The unspecific cytotoxic effect on α-sarcin (black boxes) and its derived allergen-based immunotoxin proDerp1αS (grey boxes) was evaluated on HeLa (**A**), Calu-3 (**B**), LAD2 (**C**) and Raw 264.7 (**D**) cells by MTT viability assays. Graph represents the means of duplicates and its standard deviation for all the experiments.

With the aim of testing the non-specific cytotoxic effect of proDerp1αS, somatic (HeLa and Calu-3) and immune (LAD2 and Raw 264.7) representative cell lines were incubated for 24 h with increasing tenfold concentrations of α-sarcin or proDerp1αS proteins (Fig. 4). In all cases, the cytotoxic effect of wild-type α-sarcin was significantly higher than that of the construct, most especially at the highest protein concentration assayed (1 μM) (Fig. 4). In agreement with this observation, the viability of the cultures was almost always over 90% independently of the proDerp1αS concentration assayed. The exception was Calu-3 cells, that presented a remarkable decrease in viability by proDerp1αS—already at 100 nM, but indistinguishable from the fungal natural toxin effect (Fig. 4B), suggesting therefore a non-specific origin.

### Serum sensitized humRBL-2H3 activation and viability inhibition

**Figure 5:**
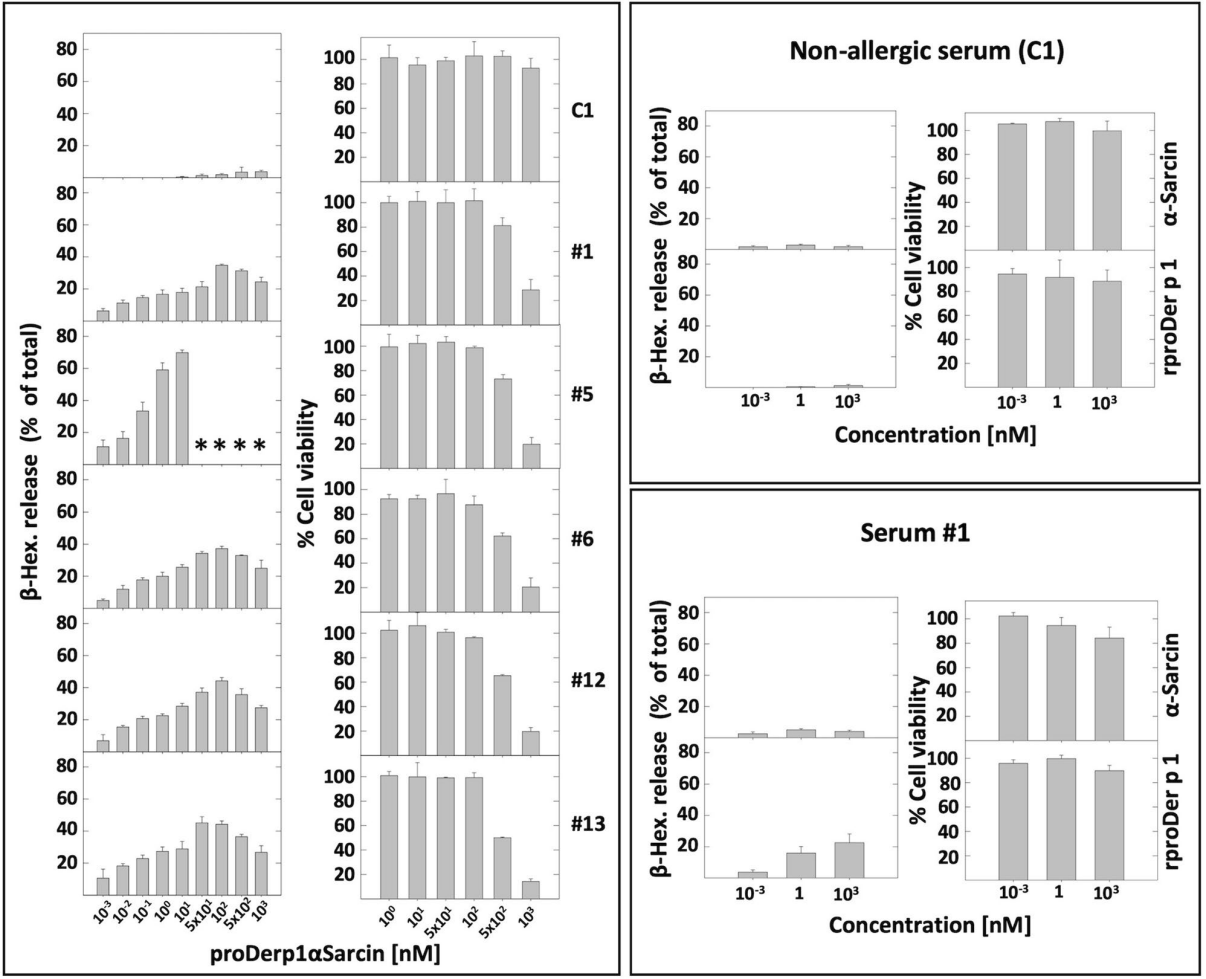
Serum sensitized humRBL-2H3 degranulation and viability inhibition assays. HumRBL-2H3 cells sensitized with Der p 1-positive allergic (#1, 5, 6, 12, 13) and non-allergic (C1) individuals’ sera were treated with proDerp1αS for degranulation and viability analysis, performed by β-hexosaminidase release (β-Hex. % of total) and MTT viability assays, respectively. α-Sarcin and rproDer p 1 were used as controls with a representative serum of each allergic and non-allergic groups (serum #1 and C1, respectively). Bars represent means of duplicates and its standard deviation for all the experiments. (*) indicates technical limitations by no longer availability for serum from this patient.

Degranulation rate of Der p 1 serum sensitized-humRBL-2H3 cells (n = 6), measured 1 h after stimulation, was similar for rproDerp1 and proDerp1αS (Fig. 5, compare results corresponding to serum #1). The β-hexosaminidase release assay manifested an allergen bimodal dose dependent effect, since after showing and increment, degranulation was again reduced at the higher protein concentrations employed, basically above 500 nM. HumRBL-2H3 activation was exclusively observed when cells were previously sensitized with HDM allergic patient sera. Supporting the specificity of this effect, α-sarcin never caused degranulation, independently of the serum treatment used (Fig. 5).

The specificity of the cytotoxic effect caused by proDerp1αS on sensitized humRBL-2H3 cells was analyzed in parallel experiments using 3-(4,5-dimethylthiazol-2-yl)-2,5-diphenyltetrazolium bromide (MTT) cell viability assays. In this case, the chimeric molecule showed high specific cytotoxicity, promoting cell death exclusively on humRBL-2H3 cells sensitized with Der p 1-allergic patients’ sera, but not on those treated with non-allergic patients’ sera (Fig. 5). Comparatively, neither rproDer p 1 nor α-sarcin showed significant cytotoxicity on Der p 1-sensitized and non-sensitized humRBL-2H3 cells, thus validating the specificity of the allergen-toxin construction.

### Human basophils degranulation

**Figure 6:**
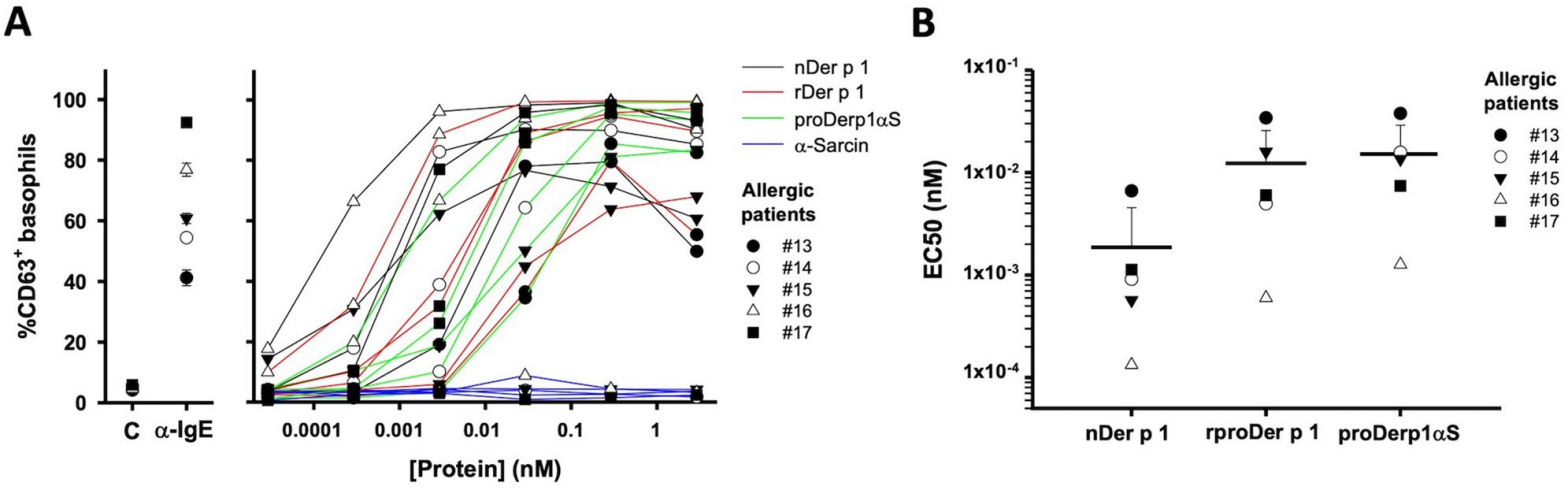
Basophil degranulation. (**A**) Basophil activation test dose dependant profiles for nDer p 1, rproDer p 1, proDerp1αS and α-sarcin performed with basophils from Der p 1-allergic patients (n = 5). Individual profiles of each patient are presented in Supplementary Figure S3. (**B**) nDer p 1, rproDer p 1 and proDerp1αS individual and medium EC_50_ representation for the five Der p 1 allergic patient. Black horizontal gross bars represent the EC_50_ means while upward deviation bars the sample standard deviation.

The biological relevance of proDerp1αS was studied in terms of degranulation using basophils from HDM allergic and non-allergic patients (Fig. 6). Basophil activation test (BAT) showed a similar trend in degranulation caused in a dose-dependent manner by nDer p 1, rproDer p 1 and proDerp1αS in the case of HDM allergic patients (n = 5), with no degranulation caused by α-sarcin in any patient analyzed (Fig. 6A). Non-allergic patients (n = 5) did not show any different activation effect respect to the controls (data not shown). According to the half maximal effective concentration promoting degranulation (EC_50_), nDer p 1 (0.00187 ± 0.00267 nM) manifested an activity in the order of tenfold higher than those of rproDer p 1 (0.0123 ± 0.0134 nM) and proDerp1αS (0.0151 ± 0.0138 nM) (Fig. 6B). Between rproDer p 1 and proDerp1αS EC_50_ median values statistically significant differences were not appreciated after the corresponding ANOVA analysis (*P* < 0.186).

### Cytotoxic effect on basophils and IgE internalization after proDerp1αS stimulation

**Figure 7:**
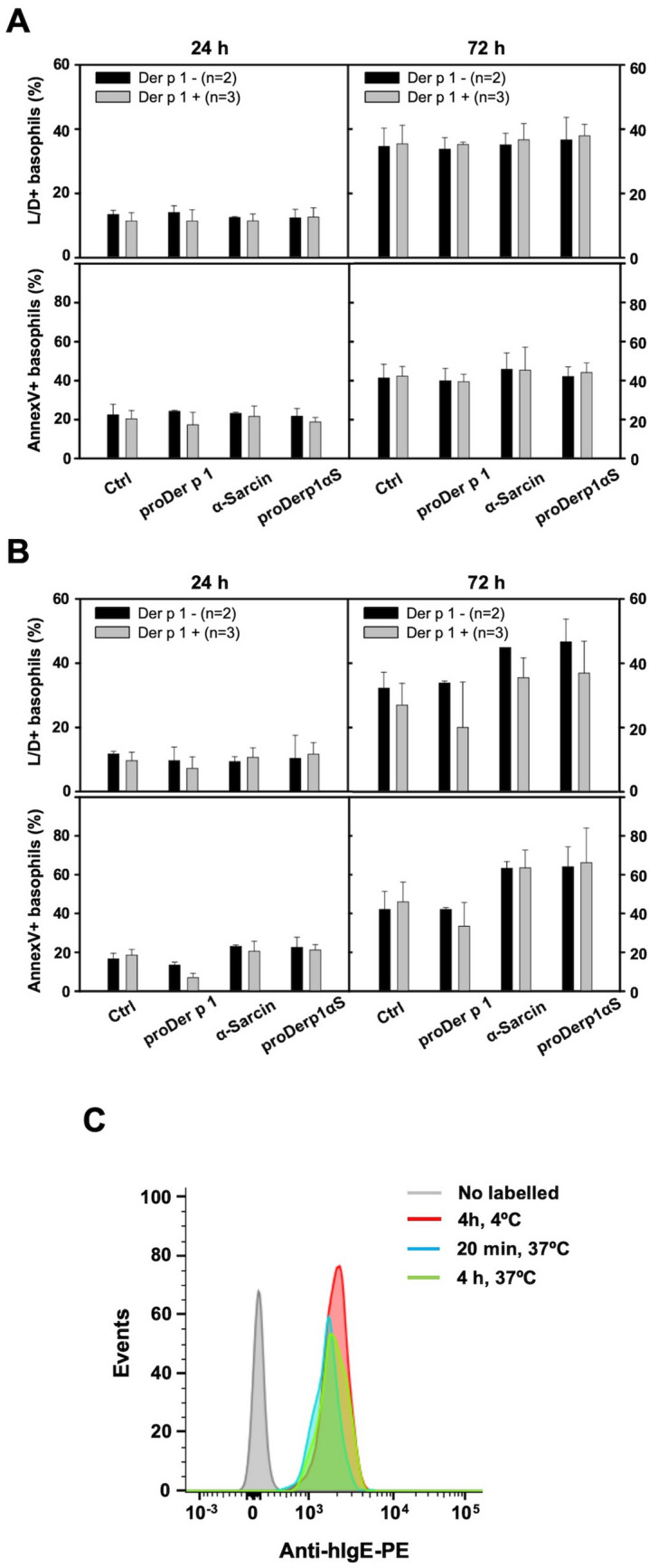
proDerp1αS cytotoxic and IgE internalization effect on basophils. Proapoptotic (annexinV-positive cells) and viability inhibiting (LIVE/DEAD-positive cells) effect of proDer p 1, α-sarcin and proDerp1αS on human basophils from Der p 1 allergic (grey boxes) and non-allergic patients (black boxes). Graphs show the results according to the protein concentration stimulus, 10 nM (**A**) and 1 μM (**B**), and incubation time, 24 or 72 h. All results include medium ± standard deviation of values obtained from three Der p 1-allergic patient and two non-allergic individuals. (**C**) Surface anti-IgE labelling on basophils (CCR3^+^/CD203c^+^) contained within different blood aliquots from patient #17 (Der p 1-allergic). After 10 min of stimulation at 37 °C with proDerp1αS, prior to external IgE labelling, the corresponding aliquots were incubated as follows: 4 h at 4 °C as non-internalization control (red), 20 min at 37 °C (blue) or 4 h at 37 °C (green). A non-labelled aliquot was included as a blank (grey).

After showing that proDerp1αS binds to specific-Der p 1 IgEs on basophils surface, with a similar behavior to that observed for nDer p 1, its cytotoxic effect on these cells was studied. As it is shown in Fig. 7A, 10 nM proDerp1αS treatment did not promote any significant increase in annexin V (apoptotic) or LIVE/DEAD (non-viable) basophil labelling respect to the non-treated cells, neither after 24 h nor after 72 h of incubation. Coherently, identical results were observed for 0.1 nM concentrations (data not shown). Higher proteins amount, up to 1 μM and after 72 h of incubation with either free wild-type α-sarcin or proDerp1αS, caused a significant increase in the percentage of apoptotic and non-viable basophils, with independence of patient sensitization subset (Fig. 7B). The assays analysing IgE-FcεRI complex internalization after proDerp1αS binding to IgE showed that basophils surface IgE levels did not change significantly in the next 4 h after immunotoxin stimulation of Der p 1-sensitized patients’ basophils (#17) (Fig. 7C).

Thereby, according to our results, due to its lack of internalization after surface IgE-binding, proDerp1αS did not produced any specific cytotoxic effect on Der p 1-sensitized basophils, regardless of incubation time (24 or 72 h) or protein concentration (0.1, 10, 1,000 nM) assayed; suggesting a much more complex scenario of primary cells instead of the previously described experiments with humRBL-2H3 cell line cultures.

## Discussion

Adequate prevention of allergies, diagnosis and treatment are one of the great health challenges of the twenty-first century. Due to the variable effectiveness among patients and sometimes adverse secondary effects of AIT^32^, new therapeutic alternatives and complementary approaches to AIT have been developed in the last years^33^. Many of them are based on biological treatments, including immunomodulatory strategies such as IgE-blocking antibodies as Omalizumab^34^ and those directed to membrane-linked IgEs (mIgE) on memory B cells^35^ and against FcƐRI receptor on effector cells^36,37^.

In this work we present a new alternative approach based on the production, and structural and functional characterization of proDerp1αS, an allergen-directed immunotoxin targeted against IgE- and/or FcεRI-positive effector cells. This proDerp1αS chimera was produced as a homogeneous soluble polypeptide chain of 53 kDa, which integrates the ability to bind FcεRI-positive cells and the cysteine protease activity described for Der p 1 allergen and the ribonucleolytic catalytic activity of α-sarcin. Although proDer p 1 has been described as an inactive zymogen due to the blocking of the active site by its N-terminal pro-peptide, proDerp1αS presented proteolytic activity against the Boc-QAR-AMC substrate, albeit minor than nDer p 1 mature protease. An explanation to this fact could rely on a structural distortion of N-terminal pro-peptide induced by the α-sarcin domain, as in proDer p 1 three-dimensional structure the C-terminal segment of the pro-peptide is located very close to proDer p 1 C-terminus, where α-sarcin is fused in the construct^18,38^.

As a major rule, the cytotoxic specificity of immunotoxins depends on the proper functionality of its marker domain^9^. In this case, this function lies on the proDer p 1 domain, which is expected to direct proDerp1αS against the allergen-specific effector cells. Therefore, its correct functionality is defined based on its adequate recognition by Der p 1-allergic patients IgEs. Accordingly, all sera from 15 allergic individuals recognized proDerp1αS in a very similar way as nDer p 1 or rproDer p 1, being the inhibition ability of the chimera slightly lower than the corresponding to nDer p 1. This was expected because of the molecular differences existing between rproDer p 1, unprocessed and non-glycosylated in Asn132, and the natural form of Der p1 (nDer p 1) to which allergic patients have been naturally sensitized^18,21,38^. These results, together with the enzymatic characterization of proDerp1αS, suggested an optimal conformational features for the immunotoxin and, more notably, an important maintenance of the main IgE-binding regions presented on nDer p 1.

proDerp1αS produced a dose-dependent degranulation on both allergic sera-sensitized humRBL-2H3 cells and Der p 1 allergic patient-derived basophils, but not on those humRBL-2H3 cells sensitized with control non-atopic sera or basophils from non-allergic individuals. These results indicate that proDerp1αS effectively binds to the surface receptors of these cells and, it occurs specifically through the IgE-FcεRI complex which cause the cross-linking of FcεRI receptors and ultimately the cell degranulation^12^.

Simultaneously, it was demonstrated that the cytotoxic effect of proDerp1αS on humRBL-2H3 cells was specific and strictly dependent on the presence of Der p 1-specific IgE on their cell surface, inhibiting cell viability only in those cells previously sensitized with Der p 1 allergic patients’ serum. In these cases, although the concentration to promote a significant decrease in cell viability was high (IC_50_ > 0.1 μM), when tested on 1 μM proDerp1αS promoted a potent cell viability inhibition, reaching values of 75–80% for all cases tested. These experiments reaffirmed the selective toxicity of proDerp1αS, showing no significant effect on the viability of the humRBL-2H3 cell cultures sensitized with control non-allergic sera.

Unlike the humRBL-2H3 cells, proDerp1αS did not produce any specific cytotoxic effect on basophils of patients allergic to Der p 1. Since the binding of proDerp1αS to these cells was previously proven, the analysis of this outcome suggested three possible explanations: (1) the lack of cell internalization of the proDerp1αS-IgE-FcεRI complex, (2) the degradation of the toxic domain of the construct before its release to the cytoplasm and (3) the resistance of basophils to the cytotoxic mechanism of ribotoxins. Since the last two options have not been previously demonstrated in any cell kind^9,26^, the most feasible justification is the absence of IgE-FcεRI internalization in basophils after their allergen-mediated cross-linking in the plasma membrane. As a result, the radically divergent behavior of proDerp1αS specific cytotoxicity between humFcεRI RBL-2H3 cells and human basophils would be based on differences in the destiny of the cellular IgEs after allergen binding on those cells. In this sense, RBL-2H3 cells have been described to suffer a rapid internalization of these allergen-IgE-FcεRI complexes^39,40^, similar to which takes place in tissue mast cells^41^. On the other hand, although human basophils have shown to internalize serum IgE through FcεRI in an allergen-independent way^42^, this internalization has not been demonstrated after allergen binding; which is in line with the current understanding of circulating basophils as purely effector cells^43,44^. As an approximation to proDerp1αS-IgE-FcεRI complexes behavior in basophils of Der p 1 allergic patients, we showed that cell membrane IgE levels remained unchanged for at least 4 h after stimulation with the immunotoxin. This result, coupled with the absence of any specific cytotoxic effect during the 72 h following stimulation with proDerp1αS, suggests that circulating human basophils do not internalize the allergen once it is bound to IgE, holding the whole complex at the surface for longer periods of time, thus preventing from the cytotoxic effects coming from proDerp1αS.

Regarding the in vivo potential effect of proDerp1αS, mIgE-Der p 1 + memory B cells—producers of specific soluble IgEs—have been proposed as one of the most relevant alternative cellular targets of our strategy, whose elimination would provide a greater therapeutic impact on the course of the allergic disease^45^. In this case, these cells present an efficient internalization of natural allergens after their binding to mIgE (membrane-bound IgEs)^46,47^, providing a promising target for proDerp1αS cytotoxic actions. Furthermore, as bolus administration of therapeutic immunotoxins provides a potential systemic range of action^8,9^, these kind of molecules would allow memory cells depletion with independence of its tissue location.

In conclusion, proDerp1αS reveals itself as a suitable proof of concept example of an allergen-directed immunotoxin. Thus, the results presented here are consistent with the potential benefit of using a new type of biologic tools capable of provoking specific cell death on those involved in allergic reactions. Although this chimeric molecule could be considered as a potent cytotoxic agent against allergy-affecting cells, there is a great need of a deep in vivo characterization of its effect during the course and allergic reaction, in order to state categorically their therapeutic potential. In short, the existence of a wide set of molecular targets which are potentially suitable for the development of more immunotoxins against allergies^48^, the increasing relevance of therapeutic antibodies in the treatment of allergic diseases^49^ and the excellent properties of these molecules in terms of specificity and cytotoxic potency^8,9^, make immunotoxins a family of biotechnological tools with great potential for the treatment of allergic inflammatory reactions.

## Methods

### proDerp1αS cloning in pPICZαA and electroporation of BG11 *P. pastoris* cells

Optimized proDer p 1 cDNA sequence for *P. pastoris* expression was acquired from IDT Technologies (Coralville, IA, USA). *Eco*RI and *Not*I restriction sites were included in 5′ and 3′ ends. After digestion, proDerp1-specific cDNA was cloned downstream of α-factor signal peptide, providing the effective secretion of the protein to the extracellular media after its being processed by kex2 protease, and upstream of α-sarcin sequence in vector pPICZαA. Sequence identity was analyzed by sequencing the whole construct including the 3-amino acids linker (Gly-Gly-Arg) between the two domains. *Pme*I-linearized pPICZαA/proDerp1αS construct was electroporated in *P. pastoris* BG11 strain. The resulting electroporation mixture was plated in YPDS-agar containing 100, 400 or 750 μg/mL of zeocin for selecting purposes.

### proDerp1αS expression and purification

proDerp1αS optimal expression was studied in different *P. pastoris* transformants. The two most productive colonies were chosen for the scaling up process. The selected colony was grown, from a preculture including zeocin, in 6 × 2 L baffled flasks containing each one 380 mL of BMGY (buffered media for yeast containing glycerol) for 24 h at 30 °C and vigorous shaking. The induction was done in 5 flasks with 200 mL of BMMY (buffered media for yeast containing methanol) induction medium during 48 h at 25 °C. After 24 h 0.5% (v/v) of methanol was added to the media. For its purification the supernatant medium was obtained by centrifugation. Then, it was dialyzed in 25 kDa membranes against 50 mM sodium phosphate buffer, 0.1 M NaCl, pH 7.5. This medium was loaded in a Ni^2+^-NTA chromatography column (HisTrapTM FF Columns, GE Healthcare, Fairfield, CT, USA). After washing the column with dialysis buffer and the same buffer containing 20 mM imidazole, the protein was eluted when adding 250 mM imidazole to the same phosphate buffer.

### Western blot analysis

After purification proceeding, the samples were examined by 15% (v/v) sodium dodecyl sulphate–polyacrylamide gel electrophoresis (SDS-PAGE). The structural identity of the purified chimera was analyzed in Western blot by both anti-Der p 1 and anti-α-sarcin rabbit polyclonal antisera and then detected with a goat anti-rabbit antibody conjugated with horseradish peroxidase (GAR-HRP).

### Mass spectrometry analysis

The molecular mass analysis by MS-MALDI TOF was carried out in Proteomics and Genomics Facility (CIB-CSIC), member of ProteoRed-ISCIII network. The experiments were performed on an Autoflex III MALDI-TOF-TOF instrument (Bruker Daltonics, Bremen, Germany) with a smartbeam laser. Samples were analyzed in the positive ion detection and delayed extraction linear mode. Typically, 1,000 laser shots were summed into a single mass spectrum. External calibration was performed, using Bovine Serum Albumin from Sigma, covering the range from 20,000 to 70,000 kDa. A saturated solution of sinapinic acid in 3:1 water/acetonitrile and 0.1% TFA was used as the matrix. The sample solution and the matrix were premixed (1:1, v/v) and then 1 µL of the mixture was spotted on the stainless steel target (Bruker-Daltonics) and allowed to dry at room temperature.

### Cysteine protease activity

Immunotoxin Der p 1 protease activity was studied comparatively to nDer p 1, through Boc-QAR-AMC (N-tert-butoxycarbonyl-Gln-Ala-Arg-AMC [AMC = 7-amino-4-methylcoumarin]) hydrolysis assay, which is widely used for the determination of the proteolysis against a peptide substrate^50^. Boc-QAR-AMC substrate (75 nM) was incubated with nDer p 1, rproDer p 1 and proDerp1αS (75 nM) in a final volume of 200 µL PBS under optimal buffer conditions (5 mM L-Cysteine) within 96-well white polystyrene microtiter plates. Peptide hydrolysis was monitored by measuring free AMC fluorescence on a FLUOStar Optima fluorometer (BMG LABTECH, Offenburg, Germany) with excitation and emission filters set at 380 nm and 480 nm, respectively, at 1 min-intervals for 10 min at 25 °C. Der p 1-free reactions were used as negative controls and background counts obtained were subtracted from each value. E64 (10 nM) cysteine protease inhibitor was included as specific activity control. All experiments were performed in duplicate.

### Ribonucleolytic activity

The specific ribonucleolytic activity of ribotoxins hydrolyzing SRL is usually detected by the release of a 400 nt fragment (α-fragment) from eukaryotic ribosomes^51^. Thereby, we studied comparatively the ribonucleolytic activity of proDerp1αS against ribosomes contained in a rabbit cell-free reticulocyte lysate, as previously described^52^. Briefly, after diluting the reticulocytes extract threefold with 40 mM Tris–HCl, pH 7.5, containing 40 mM KCl and 10 mM ethylenediaminetetraacetic acid (EDTA), 50 μL aliquots of this dilution were incubated for 15 min at room temperature with different amounts of proDerp1αS and α-sarcin as a control. The resulting RNA was analyzed following a phenol/chloroform extraction and isopropanol precipitation. Finally, after washing and resuspending the resulting pellet with 70% (v/v) − 20 °C ethanol, RNA was resuspended in DEPC-H_2_O. α-Fragment release was visualized by ethidium bromide staining of electrophoresis on denaturing 2% agarose gels.

### proDerp1αS recognition by human serum IgE

The allergen-specific IgE recognition by human sera of nDer p 1, rproDerp1 and proDerp1αS was studied by indirect ELISAs. ELISA microplate wells were coated with 37 pmol of the different proteins, binding overnight at 4 °C. Then, after blocking with 3% (w/v) non-fat milk-PBS, the selected sera, diluted tenfold in blocking buffer, were incubated for 2 h at 37 °C. Human serum IgEs were detected by using an anti-human IgE rabbit antiserum followed by a goat anti-rabbit antibody labelled with horse radish peroxidase (GAR-HRP). Between incubations, wells were washed with 0.1% (v/v) Tween 20-PBS. Development was performed by adding 100 μL/well of 0.1 M sodium citrate, pH 5.0, 4% (v/v) methanol, 3.5 mM OPD (1,2-phenylenediamine dihydrochloride), 0.16% (v/v) H_2_O_2_ and stopped with 100 μL/well of 10 N H_2_SO_4_. Specific IgE recognition was quantified by measuring the optical density at 492 nm.

Additionally, inhibition ELISA were performed for comparing IgE epitope affinity between the natural allergen, its recombinant form and the derived immunotoxin. Following the indirect ELISA protocol, microplate wells were coated with nDer p 1 (37 pmol) and incubated with sera inhibited with proDer p 1, proDerp1αS and nDer p 1. This inhibition was performed by using blocking buffer for 2 h at 37 °C under vigorous shaking. The results were shown as inhibition % according to the inhibitor concentration and relativized to non-inhibited sera. All assays included duplicates for each condition.

### Cell line cultures

HeLa cells were cultured in DMEM medium supplemented with 10% of fetal bovine serum (FBS). Calu-3 bronchial epithelial cell line, derived from lung adenocarcinoma, was cultured in DMEM/Nutrient Mixture F-12 adding 10% of FBS. Both cell cultures were harvested and propagated routinely by trypsinization. Raw 264.7 cell line, mouse macrophages, was cultured in DMEM with 10% FBS. These cells were propagated by softly scraping. Rat basophilic leukemia cells (RBL-2H3) transfected with cDNA coding for the human high affinity IgE receptor chains (humFcεRI) were kindly donated by Dr. Lothar Vogel (humRBL-2H3, Paul-Ehrlich-Institut, Langen, Germany). These cells were grown in MEM supplemented with 5% (v/v) of FBS and propagated twice a week using EDTA 10 mM. Cell cultures were confluent prior to being harvested for use in experiments. Moreover, all media contained l-glutamine (300 mg/mL), 50 U/mL of penicillin and 50 mg/mL of streptomycin. Culture maintenance was performed at 37 °C in a humidified atmosphere (CO_2_:air, 1:19, v:v). The human mast cell line LAD2^53^ was kindly provided by Drs. Dean Metcalfe and Arnold Kirshenbaum (National Institute of Allergy and Infectious Diseases, NIH, Bethesda, MD, USA). Serum-free Stem Pro-34 media (Invitrogen, Carlsbad, CA, USA) contains 2 mM l-glutamine, 100 IU/mL penicillin, 100 µg/mL streptomycin and 100 ng/mL recombinant hSCF (Peprotech, London, UK).

### N-acetyl-β-D-hexosaminidase release

For activation through cross-linking to the IgE receptor, humFcεRI expressing RBL-2H3 cells were sensitized overnight with 5% (v/v) of sera from allergic patients. Next day cells were stimulated with α-sarcin, rproDer p 1 or proDerp1αS at different concentrations. To increase and stabilize the secretion of β-hexosaminidase, 50% (v/v) D_2_O was added to the Tyrode’s buffer used for the dilution of allergens^54^. For detection of the granular enzyme β-hexosaminidase, an enzymatic colorimetric assay was used as described previously^55^. Briefly, 1 h after stimulation of the cells, 30 μl of supernatant was transferred to a 96-well plate and mixed with 50 μl of substrate solution (3.5 mg/mL p-nitrophenyl-N-acetyl-β-d-glucosaminide dissolved in 40 mM citric acid, pH 4.5). In order to calculate the total β-hexosaminidase activity, cells were lysate with 100 μl of 0.1% (v/v) Triton X-100 solution and same procedure was done. The mixtures were incubated for 60 min at 37 °C. After incubation, 100 μl of glycine 400 mM pH 10.7, was added to each well, and the absorbance was measured at 405 nm. The percentage of β-hexosaminidase release was calculated as a percentage of the total β-hexosaminidase content in the cells.

### Cell viability inhibition

The ribosome inactivation exerted by ribotoxins leads to cellular death^23^. Hence, in vitro MTT viability assays are widely used for studying the effect of immunotoxins on the metabolic activity rate of different cell lines. MTT assay for quantifying as culture viability according to the formation of insoluble purple formazan crystals by metabolic active cells by reducing the MTT yellow dye. We used this method for analyzing the effect of proDerp1αS on cultures viability with little modifications according to the cell type assayed.

HeLa, Calu-3, LAD2 and Raw 264.7 cells viability assays were performed following the general methodology described for previous immunotoxins^28^. Briefly, cells were seeded into 96-well plates in culture medium for 24 h. Then, the medium was removed and 200 μl of fresh free-FBS medium containing the assayed proteins was added. After 24 h, 100 μl of medium were removed and 20 μl of 5 mg/mL MTT added per well. Finally, after 4 h of incubation, the medium was softly removed, and formazan pellets were dissolved in 100 μl of DMSO:methanol (1:1). Colorimetric absorbance was measured at 570 nm subtracting 650 nm background. Two different experiments with independent duplicates were carried out.

RBL-2H3 cells expressing humFcεRI were plated at 20,000 cells per well in 96-well plates in 100 μl of medium including 5% (v/v) of allergic and non-allergic patients’ sera. The next day, the media were removed and the assayed proteins were added at different dilutions in 100 μl of fresh medium. After 24 h, 20 μl of 5 mg/mL MTT were added per well and incubated in culture conditions for another 4 h. Finally, the media were removed and the colorimetric absorbance was measured as explained before.

### Human basophils degranulation

The BAT assays were performed, as previously described^56^, including samples from *D. pteronyssinus* allergic patients and non-allergic individuals. First, 100 μl aliquots of fresh heparinized whole blood were stimulated for 10 min at 37 °C with 20 μl of stimulation buffer. Then, the assayed proteins, diluted in PBS, were added in 100 μl at serial tenfold dilutions from 2.9 × 10^–5^ nM to 2.9 nM. Polyclonal anti-human IgE or PBS alone were used as controls. Cells were stained with anti-hCCR3-fluorescein isothiocyanate (FITC), anti-hCD203c-peridinin chlorophyll protein complex (PerCP) and anti-hCD63-phycoeritryn (PE) monoclonal antibodies, and erythrocytes were lysed. The stimulation buffer and all the antibodies used in this assay were obtained from BioLegend (San Diego, CA, USA). Basophils were gated as CCR3^+^ and CD203c^+^. Basophil degranulation was determined according to CD63 cell surface expression. All parameters were recorded with a FACSCalibur and FACScalibur software (BD Biosciences, San Jose, CA, USA). Data were analyzed with FlowJo software (BD Biosciences, San Jose, CA, USA). In all cases, the percentage of CD63-positive basophils in the control sample was below 2.5%, setting the cut-off for a positive test to 5%. Basophil allergen threshold sensitivity for the different tested proteins was analyzed by dose response curves, using as indicator the lowest allergen concentration (EC_50_) giving 50% of maximum upregulation of CD63.

### Cytotoxic effect on human basophils

Human peripheral blood mononuclear cells (PBMCs) were isolated from whole venous blood from consenting volunteers allergic (n = 3) and non-allergic (n = 2) to HDM. Per each individual, 20 mL of blood were gently added over 4 mL of ficoll-paque (Histopaque-1077, Sigma-Aldrich, St. Louis, MO, USA) and centrifuged without brake during 30 min at 400 g, R.T. PBMCs fraction was recover and washed in a fresh tube with 30 mL of 0.9% (w/v) NaCl and then centrifuged for 10 min, 300 g, R.T. After a second wash with 50 mL of the previous solution and another centrifugation in the same conditions, the cells were resuspended in 2 mL of PBS containing 1 mM EDTA and 10% (v/v) FBS. Finally, the purified PBMCs from each patient were counted, evaluating its viability by mean of trypan blue, and cultured separately in 96-round well plates containing 2 × 10^5^ cells/well in 250 μl of RPMI including 10% (v/v) FBS and the assayed stimulus (proDerp1αS, rproDer p 1 or wild-type α-sarcin) to a final concentration of 0.1, 10 or 1,000 nM.

Apoptosis and cell viability were evaluated by mean of annexin V-FITC and LIVE/DEAD staining, respectively. Annexin V-FITC is commonly used in flow cytometry to detect apoptotic cells by its ability to bind to phosphatidylserine exposed on the outer leaflet of the plasma membrane, a marker of apoptosis. LIVE/DEAD kit (Invitrogen) is a viability cell assay used in flow cytometry for simultaneous fluorescence staining of viable and dead cell, as it contains calcein-acetoxymethyl and propidium iodide (PI) solutions. In this case non-viable cells were assessed by PI staining (LIVE/DEAD+), as it intercalates with the nuclear DNA just in those dead cells with areas of disordered cell membrane.

Briefly, after incubating the cultures for 24 or 72 h, culture plates were centrifuged for 5 min at 450 g, resuspending the cells in 100 μl/well of annexin V binding buffer (50 mM HEPES, 0.7 M NaCl, 12.5 mM CaCl2, pH 7.4) containing 1.5 μg/mL of annexin V-FITC or LIVE/DEAD staining diluted 1/1,000.

After incubating 30 min at R.T., the cells were washed with 200 μl of annexin V binding buffer and analyzed in a FACScalibur apparatus (BD Biosciences, San Jose, CA, USA). Respectively, basophils were gated as CCR3^+^/CD203c^+^ within PBMCs lymphocyte/basophil region (FSC/SSC selection) by mean of anti-hCCR3-PE (BioLegend, San Diego, CA, USA) and anti-hCD203c-PerCP antibodies. Single and double staining controls were performed for compensating the overlapping fluorescence.

### Allergen-IgE-FcεRI complex internalization

Using the same general procedure as for the BAT, 100 μL of whole blood aliquots were incubated for 10 min at 37 °C after adding 20 μL of stimulation buffer. Then, proDerp1αS was added to a final concentration of 10 nM and incubated during 20 min and 4 h at 37 °C or directly in ice for non-internalization control. After desired times, the cells were fixed and labelled with anti-hCCR3-FITC, anti-hCD203C-PerCP and anti-hIgE-PE antibodies (BioLegend, San Diego, CA, USA). Samples were tested in a FACScalibur analyzer (BD Biosciences, San Jose, CA, USA), gating on basophils as CCR3^+^/CD203c^+^, and following anti-hIgE-PE fluorescence indicative of IgE-FcεRI complex internalization.

### Patient work-up

HDM-allergic patients included in this work presented a history of persistent allergic rhinitis or/and asthma to *D. pteronyssinus*, having specific IgE levels to *D. pteronyssinus* higher than 0.35 kU/L (ImmunoCAP-FEIA). Non-allergic subject group was included as a control (specific IgE < 0.35 kU/L). Levels of specific IgE to *D. pteronyssinus* crude extract in sera from allergic patients and non-allergic donors are included in Supplementary Table S1.

### Sample collection, processing and storing

The biological samples from allergic and non-allergic patients were conducted in accordance with the Declaration of Helsinki. The study was approved by the local ethics committee (Comité de Ética de la Investigación provincial de Málaga; Servicio Andaluz de Salud, Consejería de Salud Andalucía, España). All participants signed informed consent forms before the study began.

Peripheral blood samples were collected from patients and processed immediately. PBMCs, isolated by Ficoll-Paque density gradient (Pharmacia Biotech, Barcelona, Spain), and fresh blood aliquots were treated for BAT and cytotoxicity assays as described above.

Serum was collected for specific IgE determination, and stored at − 20 °C until further use. Samples were managed by the Málaga Hospital-IBIMA Biobank that belongs to the Andalusian Public Health System Biobank.

### Specific IgE determination

Levels of specific IgE to *D. pteronissynus* were determined by ImmunoCAP-FEIA in serum samples, according to the manufacturer’s instructions (Thermo Fisher Scientific, Massachusetts, USA).

### Statistical analysis

ANOVA with a post hoc analysis by the Student–Newman–Keuls’ test was used, within each test, to compare the results obtained with the different constructions for each concentration in the different assays. All values were expressed as arithmetic means ± sem (standard error of the media). Differences between experimental groups were considered statistically significant at *P* < 0.05.

### Equipment and settings

Gel image from Figs. 1B and 2B was acquired and analyzed using the Gel Doc XR Imaging System provided with Quantity One 1-D v4.6 analysis software (Bio-Rad, Hercules, CA, USA; www.bio-rad.com). Blots images from Fig. 1B, were acquired and analyzed using UVP ChemiDoc-It^TS2^ provided with UPV VisionWorks LS 7.0 analysis software (www.uvp.com). No high-contrast gels or blots has been displayed. When processing of brightness and contrast of gel and blot images has been made it was applied to the entire image including controls. No cropped gels neither juxtaposing images were displayed.

SigmaPlot-11.0 Scientific Data Analysis and Graphing Software (Systat Software Inc.; www.sigmaplot.co.uk) was used for statistical analysis and graphing of experimental data in Figs. 1C, 2A, 3, 4, 5, 6 and 7.

Flow cytometry data were analyzed with FlowJo v10.6.2 software (BD.Biosciences, San Jose, CA, USA; www.flowjo.com).

## Data availability

All experimental data generated or analyzed during this study are included in the article.

## Supplementary information

Supplementary Information 1.
