# Supplementary material for: Der p 1-based immunotoxin as potential tool for the treatment of dust mite respiratory allergy
Source: Sci Rep. 2020 Jul 23;10:12255. doi: 10.1038/s41598-020-69166-w (PMC7378242; doi:10.1038/s41598-020-69166-w)
Supplement: Supplementary file 1 — Supplementary Information 1. [file 41598_2020_69166_MOESM1_ESM.pdf]

Der p 1-based Immunotoxin as Potential Tool for the Treatment of Dust Mite respiratory Allergy

Lázaro-Gorines, R.
López-Rodríguez, JC.
Benedé, S.
González, M.
Mayorga, C.
Vogel, L.
Martínez-del-Pozo, A.
Lacadena, J.
Villalba, M.

Figure S1

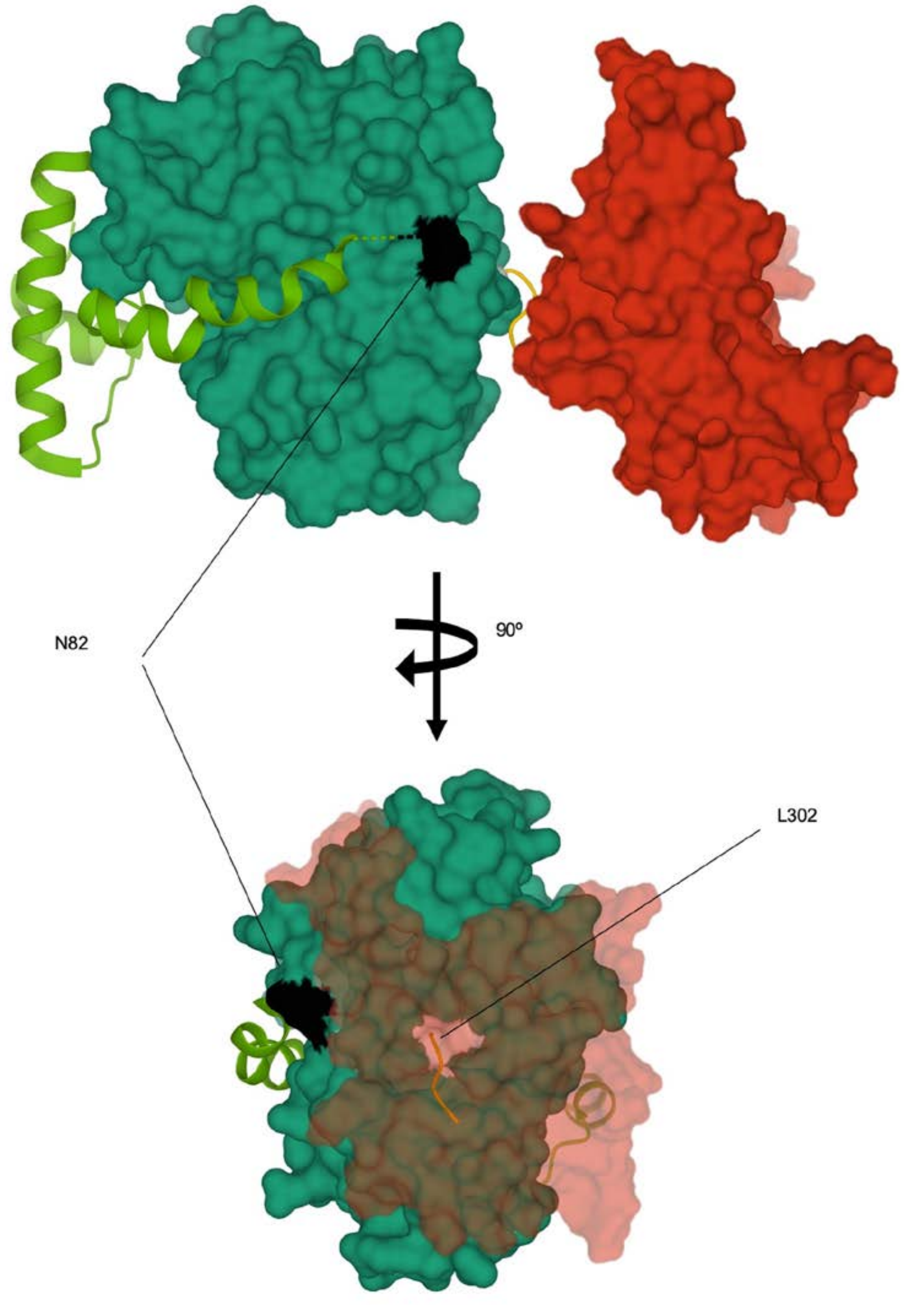

Figure S1

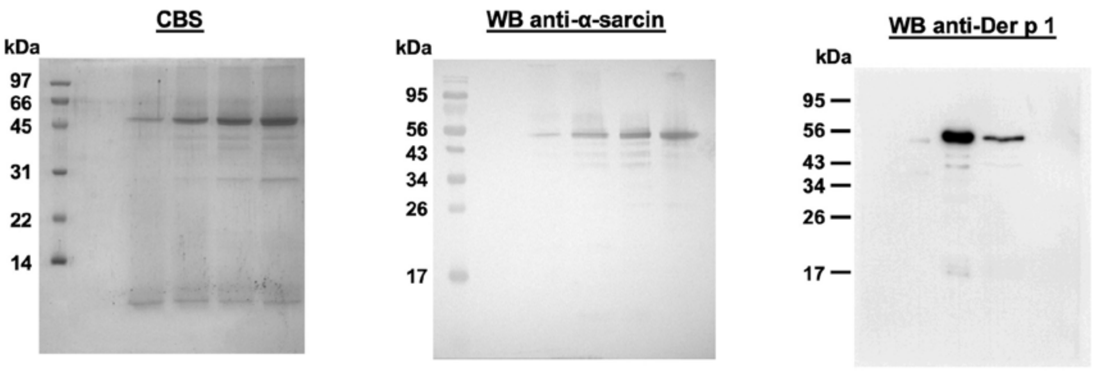
CBS
kDa
97
66
45
31
22
14
WB anti-α-sarcin
kDa
95
56
43
34
26
17
WB anti-Der p 1
kDa
95
56
43
34
26
17

Figure S2

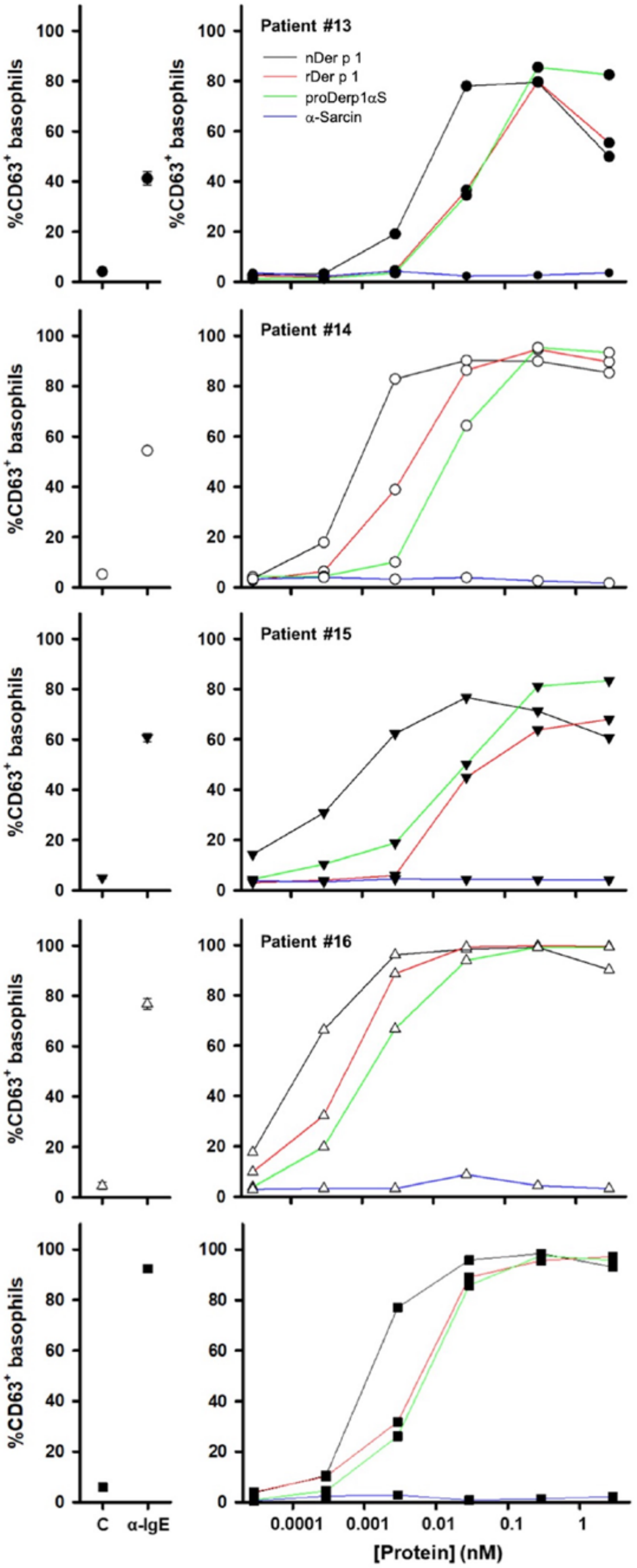
Patient #13
nDer p 1
rDer p 1
proDerp1αS
α-Sarcin
Patient #14
Patient #15
Patient #16
%CD63+ basophils
C
α-IgE
[Protein] (nM)
0.0001
0.001
0.01
0.1
1

Figure S3

Serum number	sIgE to *D. pteronyssinus* (kU/L)
1*	57.5
2	47.7
3	36.2
4	76.7
5*	100
6*	84.3
7	51.4
8	36.2
9	47.4
10	100
11	100
12*	30.3
13*/**	>100
14**	>100
15**	27.3
16**	51,1
17**	>100
C1*	<0.35
C2**	<0.35

Table S1

Supplementary Figure Legends

Figure S1. Schematic representation of proDerp1αS immunotoxin structure. Three-dimensional conformational representation of proDerp1αS construct built in accordance to crystal structures of Der p 1 pro form (PDB code: 1XKG; in turquoise) and α-sarcin (PDB code: 1DE3; in red). The green ribbon diagram represents Der p 1 N-terminal pro-peptide while the glycine-glycine-arginine linker (GGR) appears in yellow. The structures of mature Der p 1 and α-sarcin are shown as molecular surface representations. The Der p 1 mature N- and C-terminal residues (N82, in black, and L302, in white) are also indicated.

Figure S2. Coomassie blue Staining (CBS) SDS-PAGE and Western Blot analysis of purified proDerp1αS immunotoxin. Full-length gels from Figure 1B are displayed. CBS SDS-PAGE (left) and Western blot analysis using rabbit anti-α-sarcin (middle) or anti-Der p 1 antisera (right). CBS protein molecular weight standards correspond to Bio-Rad Unstained SDS-PAGE low range Standards; while those shown in the blots are prestained Bio-Rad Precision Plus protein molecular weight standards. Images correspond to full-length gels and blots acquired and analysed using the Gel Doc XR Imaging System and Quantity One 1-D analysis software (BioRad) (left/center) or ChemiDoc-It (UVP) and VisionWorks LS (right).

Figure S3. Individual basophil degranulation profile for each patient. Basophil activation test dose dependant profiles for nDer p 1, rproDer p 1, proDerp1αS and α-sarcin performed with basophils from Der p 1-allergic patients (n=5; #13, 14, 15, 16 and 17). Non-stimulated (C) and α-IgE antibody stimulated samples (α-IgE) were included as controls in each patient test (left column plots). Overlapped and analysed data are displayed in Figure 6.

Table S1. Levels of specific IgE to *D. pteronyssinus* crude extract in sera from donors. Specific IgE (sIgE) level from allergic patients (#1-17) and non-allergic subjects (C1 and C2) was determined by ImmunoCAP-FEIA in serum samples. Single asterisk indicates sera used in *in vitro* humRBL-2H3 degranulation assays and viability assays. Black arrows indicate for *in vitro* humRBL-2H3 degranulation and viability assays. Double asterisk indicates selected patients for BAT and basophils cytotoxicity assays.
